# Electrical muscle stimulation mobilizes endothelial progenitor cells in ICU patients

**DOI:** 10.1186/2197-425X-3-S1-A207

**Published:** 2015-10-01

**Authors:** C Stefanou, G Mitsiou, E Karatzanos, K Psarra, E Angelopoulos, S Dimopoulos, V Gerovasili, I Vasileiadis, C Routsi, S Nanas

**Affiliations:** National and Kapodistrian University of Athens, First Critical Care Departement, Athens, Greece; Evangelismos General Hospital, Immunology-Histocompatibility, Athens, Greece; National and Kapodistrian University of Athens, First Department of Respiratory Medicine, Athens, Greece

## Intr

In the critically ill, the number of the bone marrow-derived endothelial progenitor cells (EPCs) in peripheral blood constitutes a regeneration index of the endothelium of tissues that suffered the consequences of critical illness. Furthermore, electrical muscle stimulation (EMS) has been shown to induce beneficial effects in relation to prevention of acquired muscle weakness and atrophy, as well as weaning duration. The acute effects of EMS on EPCs have been scantily explored.

## Objectives

Our hypothesis was that EMS, an exercise equivalent, increases EPCs in ICU patients.

## Methods

Sample was consisted of 32 mechanically ventilated (for >72 hrs) septic patients of a multidisciplinary ICU, aged (mean ± SD) 58 ± 14 yrs. Ten patients were on steroids (group S), while 22 patients were not (group A) (APACHE-II score: 21.5 ± 8.7 vs 19.6 ± 7.2 respectively). Patients were randomized to one of two 30 min EMS biphasic protocols (PR1: 75 Hz, 400µsec, 6s on - 21s off, PR2: 45 Hz, 400µsec, 5s on - 12s off). Blood was sampled before and immediately after the EMS implementation. EPCs were quantified by flow cytometry, utilizing the surface markers CD34, CD133, CD45; for the mature circulating endothelial cells (CECs, indices of endothelial injury), CD34, CD45, VEGFR_2_ were utilized.

## Results

In all patients, EPCs (cells / million enucleated, mean ± SE) increased from 13.53 ± 1.80 to 20.81 ± 2.99 (p = 0.013). Group S demonstrated a different response from group A (p = 0.015). In group A, EPCs increased from 12.09 ± 2.27 to 23.82 ± 4.05 (p = 0.002). In group S, EPCs did not change (from 16.70 ± 2.79 to 14.20 ± 2.69, p = 0.46). PR1 did not differ from PR2 (p = 0.60 in whole cohort, p = 0.67 in group S, p = 0.84 in group A). No correlation among the EPCs increase and the contraction force (r = 0.24, p = 0.28) or maximal current (r = 0.12, p = 0.64) was observed. Furthermore, in all patients CECs increased from 16.50 ± 2.57 to 23.84 ± 3.40 (p = 0.008). No difference between the groups A and S (p = 0.21) or the EMS protocols PR1 and PR2 (p = 0.20) was found. During EMS, no side effects, ECG or haemodynamic complications were observed.

## Conclusions

The present study showed that the number of EPCs and CECs increases after EMS implementation in septic, critically ill patients. No difference was established between the two EMS protocols. Steroids, likely because they are marrow suppressants, seem to inhibit EPCs. EMS may act beneficially by mobilizing EPCs, in the same direction as exercise. Further investigation is required to explore the underlying pathophysiological mechanism.
